# Design and Mechanical Properties Verification of Gradient Voronoi Scaffold for Bone Tissue Engineering

**DOI:** 10.3390/mi12060664

**Published:** 2021-06-05

**Authors:** Haiyuan Zhao, Yafeng Han, Chen Pan, Ding Yang, Haotian Wang, Tingyu Wang, Xinyun Zeng, Penglei Su

**Affiliations:** 1 School of Mechanical Engineering, Beijing Institute of Technology, Zhongguancun South Street No.5, Haidian District, Beijing 100081, China; 3120180429@bit.edu.cn (H.Z.); 3220195044@bit.edu.cn (C.P.); 3120190412@bit.edu.cn (D.Y.); 3220190208@bit.edu.cn (H.W.); 3220190210@bit.edu.cn (T.W.); 3220200313@bit.edu.cn (P.S.); 2 Institute of Engineering Medicine, Beijing Institute of Technology, Zhongguancun South Street No.5, Haidian District, Beijing 100081, China; 3220201944@bit.edu.cn

**Keywords:** Voronoi-tessellation, irregular porous structure, gradient bionic scaffold, stress shielding, stability verification, impact resistance verification

## Abstract

In order to obtain scaffold that can meet the therapeutic effect, researchers have carried out research on irregular porous structures. However, there are deficiencies in the design method of accurately controlling the apparent elastic modulus of the structure at present. Natural bone has a gradient porous structure. However, there are few studies on the mechanical property advantages of gradient bionic bone scaffold. In this paper, an improved method based on Voronoi-tessellation is proposed. The method can get controllable gradient scaffolds to fit the modulus of natural bone, and accurately control the apparent elastic modulus of porous structure, which is conducive to improving the stress shielding. To verify the designed structure can be fabricated by additive manufacturing, several designed models are obtained by SLM and EBM. Through finite element analysis (FEA), it is verified that the irregular porous structure based on Voronoi-tessellation is more stable than the traditional regular porous structure of the same structure volume, the same pore number and the same material. Furthermore, it is verified that the gradient irregular structure has a better stability than the non-gradient structure. An experiment is conducted successfully to verify the stability performance got by FEA. In addition, a dynamic impact FEA is also performed to simulate impact resistance. The result shows that the impact resistance of the regular porous structure, the irregular porous structure and the gradient irregular porous structure becomes better in turn. The mechanical property verification provides a theoretical basis for the structural design of gradient irregular porous bone tissue engineering scaffolds.

## 1. Introduction

There are 15 million cases of bone fracture in the world every year, more than 3 million cases of bone injury [1]. According to the relevant literature statistics [2], the incidence of bone nonunion after fracture treatment is up to 20%. There are two main reasons for bone nonunion: biological factors and mechanical factors. Severe fracture injuries result in excessive destruction of bone tissue at the fracture site and adjacent bones. After severe fractures, such as comminuted fractures, a part of the bone might be lost. The mechanical properties of fracture are unstable, so the fracture end deformation and displacement are easily caused by the change of stress direction at the fracture end. In order to reduce the probability of nonunion and reduce the risk of secondary surgery, osteogenic and osteoinductive substances must be supplemented to shorten the healing time and promote fracture healing and achieve the purpose of surgical treatment. Bone graft is the most widely used and mature method to treat fracture loss and promote bone healing. Autogenous bone graft is still the gold standard approach when dealing with orthopedic trauma or disease in bone graft [1]. However, due to the limitation of autogenous bone supply and the secondary trauma caused by surgery, bone tissue engineering scaffolds have been widely used in bone replacement and bone treatment for severe fracture defects [3].

The development of additive manufacturing technology (AM) provides more possibilities for scaffold design methods [4]. The structure which is difficult to manufacture by traditional manufacturing methods can be obtained by AM. AM promotes the development of porous bone tissue engineering scaffolds [5]. The porous bone tissue engineering scaffold not only provides a larger surface area for the growth of new bone, but also facilitates the circulation of nutrients due to the interconnected pores [6,7].

**Figure 1:**
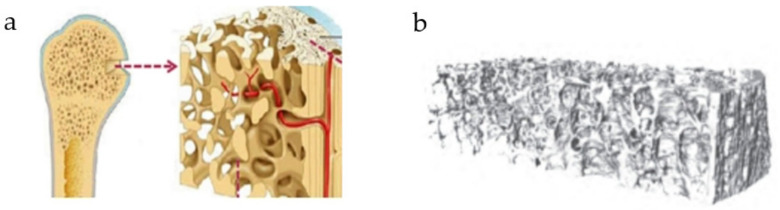
Schematic diagram of bone tissue: (**a**) Hierarchical structural organization of bone; (**b**) Microstructure of human vertebra.

At present, the porous structure can be divided into regular porous structure and irregular porous structure [8]. Different pore structures will lead to different mechanical properties of scaffolds. The regular porous structure shows some periodicity. Periodic structure will lead to great changes in the overall performance of the structure when its basic units change. By observing the microscopic structure of natural bone, it is found that the structure of natural bone, especially the trabecular structure, is irregular in the microscopic structure. The apparent elastic modulus of a structure is related to its porosity [9]. Some researchers have established three-dimensional microstructures of human vertebrae [10], showing the pore structure of human bone with gradient changes in structural density and gradient changes in Young’s modulus (Figure 1). Some scholars have studied the design of porous structures and the fact that the apparent elastic modulus of structures with different porosity is different has been verified [11].

An important problem of scaffold is stress shielding. Similar modulus can reduce stress shielding [12,13]. This also provides us with an idea: if the porosity of different bone parts is properly controlled and the apparent elastic modulus of these parts are close to the human bone, the stress shielding effect between the scaffold and the natural bone can be improved. This is beneficial to stimulate the growth of bone and improve the service life of scaffolds [14]. However, there are few studies on the design methods of gradient structure at present, and how to control the gradient change of structure accurately while ensuring the irregular porous structure is a difficult point. It also remains to be proved whether irregular structure can actually improve scaffold performance.

This study presents a structural design method based on Voronoi-tessellation. This method allows us to regulate the apparent elastic modulus of different parts of the scaffold by controlling the change of structural gradient. In this study, several models were designed and manufactured by SLM and EBM. Through finite element analysis (FEA), we compared the stability and impact resistance of the gradient irregular porous structure, the irregular porous structure and the regular porous structure, and verified the stability by experiments.

## 2. Materials and Methods

### 2.1. Design Method

#### 2.1.1. Design Principle

**Figure 2:**
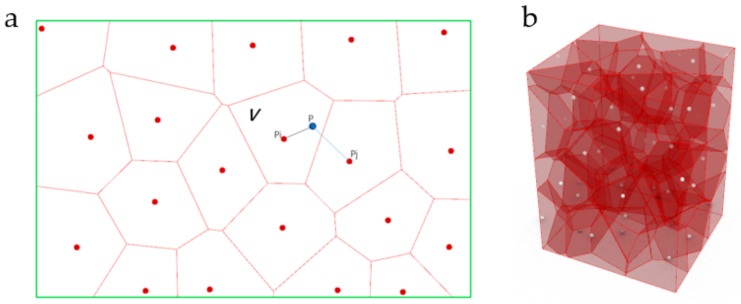
Schematic diagram of Voronoi-cells: (**a**) Two-dimensional Voronoi-cells; (**b**) Three-dimensional Voronoi-cells.

The design method of the irregular porous structure is based on Voronoi-tessellation. Voronoi-tessellation is a method of dividing spatial regions [15]. In a specific space, with different points in the space as the core. Different sub-region spaces are generated (Figure 2). Any point in the space has these characteristics:

(1) $$V\left(P_{i}\right)=\{P|D\left(P,\;P_{i}\right)\;\leq \;D\left(P,\;P_{j}\right),\;i\;\neq \;j,\;j\;=1,2,3,\;\ldots ,n\}$$

where:

- Ω is a Voronoi-cell sub-region space in a specific space;
- *P_i_* is the core of Ω;
- P is any point different from *P_i_* in Ω;
- *P_j_* is the core of other Voronoi-cell sub-regional spaces;
- D is the distance between two points;
- V stands for Voronoi-cell sub-area space.

This method can generate many irregular small polyhedral structures in the design space. The common edges of polyhedrons are used as the generatrix of the generating beam structure. In this way, complex and irregular beam structures can be generated in the design space. These beam structures are used to simulate the irregular trabecular structure of natural bone.

3D design software Rhino 6 (Robert McNeel & Associates, Headquarters, North America, and Pacific 3670 Woodland Park Ave N) (https://www.rhino3d.com/cn/, accessed on 3 May 2021) supports the design method based on Voronoi-tessellation. Grasshopper, a plug-in software of Rhino 6, can facilitate us to generate a set of controllable number of random points in the design space. With a series of random points as the core, a set of Voronoi-cells (polyhedral structures) are generated in the design space. Based on this design method, three design variables are involved in the design process: random number *R*, seed number *N* and scale factor *F*.

The apparent elastic modulus and strength of porous structure are closely related to the porosity of the structure. Gibson Ashby model can simplify irregular porous structure material into regular structure material which is composed of special basic units. The apparent elastic modulus and compressive strength of irregular porous structure can be related to the porosity of the structure. The apparent elastic modulus of the structure can be controlled by controlling the porosity of the structure [11]. The Gibson Ashby model of porous structure is as follows:

(2) $$E=k_{1}\times {\left(1-P\right)}^{m}\times E_{0}$$

(3) $$S=k_{2}\times {\left(1-P\right)}^{m}\times S_{0}$$

K_1_ and K_2_ is a parameter related to the material property itself. S_0_ is the elastic modulus of a compact material. E_0_ is the strength of a compact material.

The porosity of structure can be got by this equation:

(4) $$P=\frac{V-V_{s}}{V}\times 100\%$$

*V* is the volume of the design space. *V_s_* is the solid volume of the structure. P is the porosity of structure.

Assuming that the target average pore radius of the structure is r_0_, and the target porosity is *P*_0_, we can use the following formula to estimate the number of seeds:

(5) $$N\approx \frac{V\times P_{0}}{\frac{4\times \pi }{3}\times r_{0}{}^{3}}$$

#### 2.1.2. Design Method

In the software Grasshopper environment, we designed a set of methods to control the distribution of random points. We control the distribution of the number density of Voronoi-cells by controlling the distribution of random points, thereby controlling the density distribution of the scaffold structure and obtaining the desired structural gradient. On the basis of Voronoi-cell, a beam structure similar to trabecular bone is generated by the method of Boolean operation. In this way, we can control the smooth transition to the gradient density of the structure.

**Figure 3:**
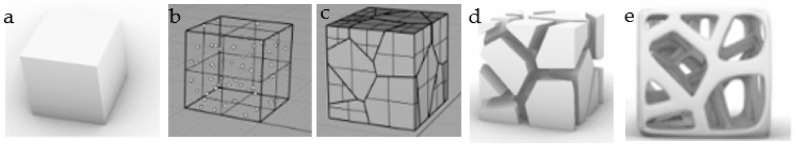
Structure design steps: (**a**) Step 1, assume the cuboid as the design space; (**b**) Step 2, randomly generate n discrete points in the cuboid; (**c**) Step 3, take discrete points as the core to generate Voronoi polyhedrons with common edges; (**d**,**e**) Step 4, get the beam structure through Boolean operation.

The method to obtain irregular porous scaffold can be simply divided into several steps (Figure 3). Step 1, assume the cuboid as the design space; Step 2, randomly generate n discrete points in the cuboid; Step 3, take discrete points as the core to generate Voronoi polyhedrons with common edges; Step 4, get the beam structure by Boolean operation. The distribution of points affects the distribution of the Voronoi polyhedrons and the solid parts, and then affects the change of the apparent elastic modulus of the structure.

#### 2.1.3. Seeds Control Method

In order to obtain the trabecular structure with pore gradient distribution similar to natural bone, controlling the distribution of core points is one of the most important things.

**Figure 4:**
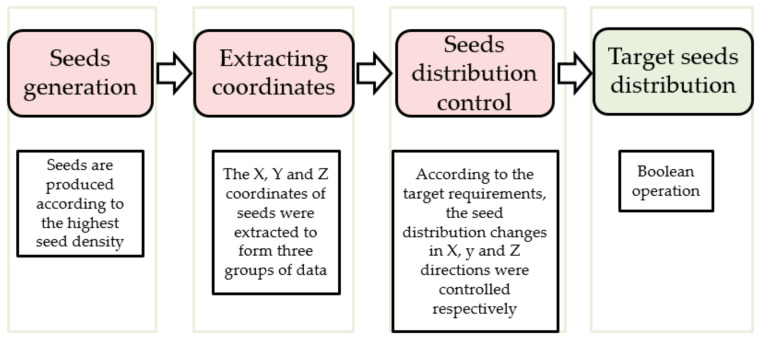
Schematic diagram of method to control points distribution.

This is the main steps of controlling seed distribution. Step 1, a certain number of seeds are generated randomly in the design space. The number of seeds generated in this step needs to be much larger than the target number N. Step 2, extract the X, Y and Z coordinates of all seeds respectively, and generate three sets of data sets. One data set contains the X coordinates of all seeds. One data set contains the Y coordinates of all seeds. One data set contains the Z coordinates of all seeds. Step 3, set expression function or graph function to control the seed distribution in X, Y and Z directions. In this step, the seeds that do not conform to the target seed distribution law are deleted by Boolean operation. Step 4, through Boolean operation, the seeds which conform to the distribution law of target seeds in X, Y and Z directions are integrated. Figure 4 is the schematic diagram of the method to control points distribution.

#### 2.1.4. Porosity Control Method

**Figure 5:**
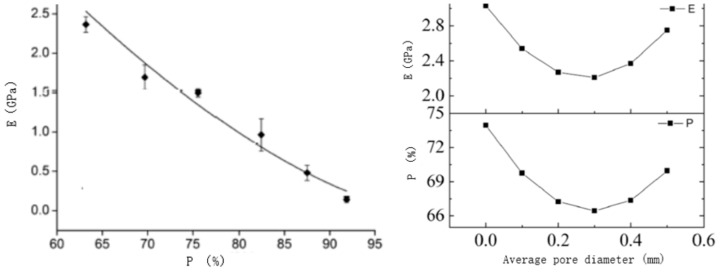
The relationship between the porosity and the apparent elastic modulus [18,19].

The mismatch of elastic modulus leads to stress shielding and affects fracture healing [16,17]. Some studies show that the design method based on Voronoi-tessellation can obtain different apparent elastic modulus of the structure by controlling the porosity of the structure [18,19]. The relationship between the porosity and the apparent elastic modulus of the structure is shown in Figure 5.

**Figure 6:**
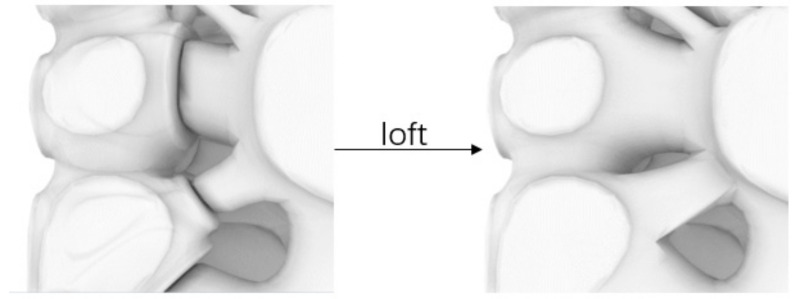
The smooth transition between solid structures after “loft”.

Therefore, the gradient distribution of porosity should be controlled to control the gradient distribution of apparent elastic modulus. It is a key point to study how to achieve different porosity by controlling variables. This will be discussed in detail in Section 3.1. Moreover, the change of porosity is accompanied by the change of solid structure. The smooth transition between solid structures also needs to be solved. After research, the “loft” module in grasshopper can realize the smooth transition between solid structures (Figure 6). There is no doubt that it is beneficial to reducing stress concentration.

### 2.2. Stability Verification Method

**Figure 7:**
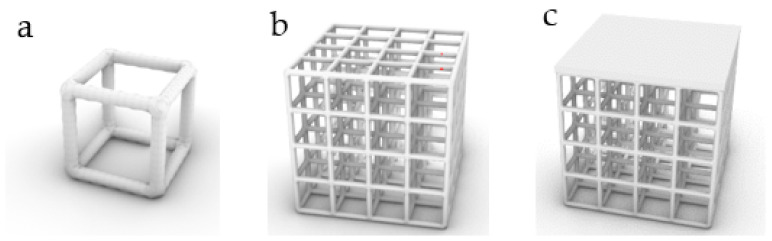
Structure 1: (**a**) Basic unit cell; (**b**) Regular porous structure; (**c**) Regular porous structure with a thin plate.

**Figure 8:**
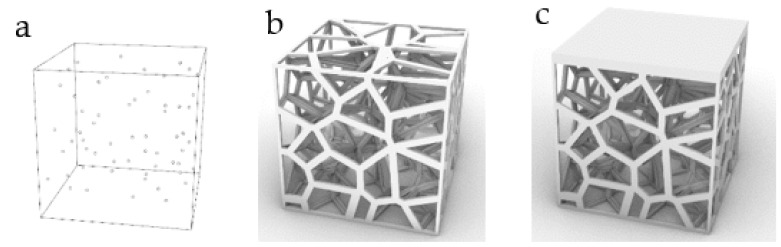
Structure 2: (**a**) Core points distribution; (**b**) Irregular porous structure; (**c**) Irregular porous structure with a thin plate.

**Figure 9:**
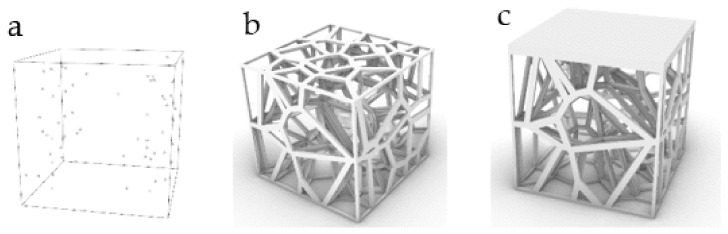
Structure 3: (**a**) Gradient core points distribution; (**b**) Gradient irregular porous structure; (**c**) Gradient irregular porous structure with a thin plate.

**Figure 10:**
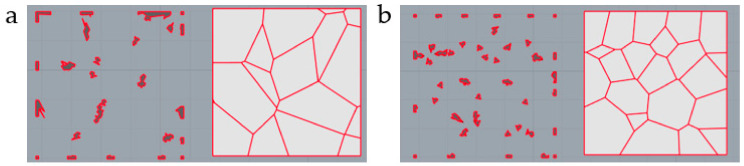
Z-axis sections of the beam structure in structure 3: (**a**) Section near the middle of the structure (The left is the section of support beams and the right is the section of Voronoi polyhedrons); (**b**) Section near the thin plate.

In order to verify the mechanical properties of irregular porous structure, a cube with sides length of 5 mm is used as the design space. In three cubes of the same size, three simplified structures are designed, respectively. In order to control other variables, the three structures have the same volume of 8.28 mm^3^ and the same seed number of 64. The porosity of the three structures is 94.4% calculated by Formula (4). In order to verify the stability of the three structures, we add a thin plate with the same thickness and volume to the upper surface of the three structures, and the thickness is 0.2 mm. Structure 1 is a representative basic regular porous structure (Figure 7). The basic unit cell structure is six intersecting prismatic structures of a cube. Structure 2 is an irregular porous structure designed by us (Figure 8). Structure 3 is an irregular porous structure with gradient changes in the direction perpendicular to the sheet surface (Figure 9). The discrete points are mainly distributed at both ends of the z-axis of the box in the design of structure 3. Beams as the internal support of structure 2, their distribution is relatively uniform, which can be seen from the distribution of discrete points at the beginning of the design. However, for structure 3, due to the distribution of points in the structure, there are more Voronoi polyhedrons near the plate than in the middle. There are more internal support beams near the plate (Figure 10).

In view of the low elastic modulus and good biocompatibility of Ti6Al4V. Ti6Al4V is often used as biomaterial material [20,21,22]. We set the parameters such as the elastic modulus of the material Ti6Al4V as the FEA parameters of the three structures.

**Figure 11:**
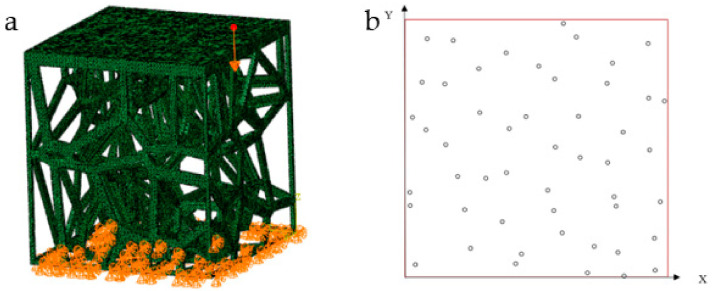
Schematic diagram of FEA: (**a**) The vertical force is applied to one of the 50 points; (**b**) Positions of 50 random points.

Considering that the maximum stress is in the normal direction when the bone is under the force, we mainly analyze the stability of the structure by comparing the displacement of the stress point of the thin plate when the structure is under the normal force. In total, 50 points are randomly generated on the surface of the thin plate. Using a vertex on the boundary of the thin plate as the coordinate origin to establish a rectangular coordinate system to obtain the coordinates of 50 random points (Table 1, Figure 11b). Give each point the same vertical force of 50 N (Considering the maximum stress of human bone during activity [23,24]). The bottom surface of the structure is fixed. Using a vertex on the boundary of the thin surface as the coordinate origin to establish a rectangular coordinate system to obtain the coordinates of 50 random stress points (Table 1, Figure 11a).

Record the final normal displacement of the point after the action of a force. Finally, the normal displacements of the stress points of the three structures of 50 different forces are collected.

### 2.3. Impact Resistance Verification Method

**Figure 12:**
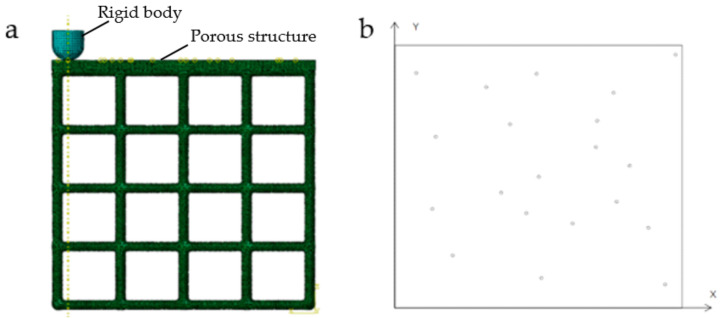
(**a**) Schematic diagram when the rigid body impacts at one of 20 positions; (**b**) Positions of 20 random points.

The above structures and materials are also used for impact resistance verification. The condition of FEA is different from the stability verification. In the verification of impact resistance, four side faces of the structure are fixed. A rigid body with a hemispherical head is used to impact the structure (Figure 12a). In total, 20 points are randomly selected on the surface of the plate as the impact positions of the rigid body (Table 2, Figure 12b). The hemispherical surface of the rigid body is 0 mm away from the structural plate at the initial position. After the rigid body applied impact on the plate, we compare the impact resistance of the three structures by comparing displacement of all points on the structural plane opposite to the plate.

**Figure 13:**
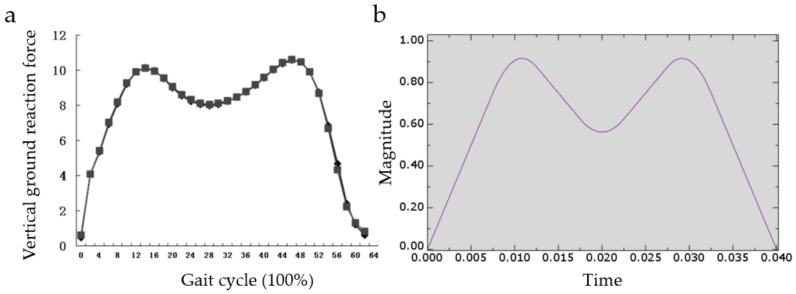
(**a**) Curve of vertical ground reaction force during natural walking; (**b**) Impact force used in FEA.

Huang Ping [25] has studied vertical ground reaction during natural walking. The curve takes the shape of M (Figure 13a). Suppose a person’s walk frequency is about 4 steps per second. Then a stress cycle is 0.5 s. We take the vertical reaction force of the ground on people’s walking as the impact force. When people are running or doing strenuous exercise, the force on the tibia is several times increased [26]. In order to make the simulation analysis more representative, we improve the force and reduce the stress period. The maximum impact force of 10 g is given to the rigid body, and the action period of the impact force is set as 0.04 s (Figure 13b). Finally, the maximum displacement and average displacement of all points on the bottom are collected to compare the impact resistance of the three structures.

## 3. Results and Discussion

### 3.1. Design Analysis

#### 3.1.1. Analysis of Design Variables

**Figure 14:**
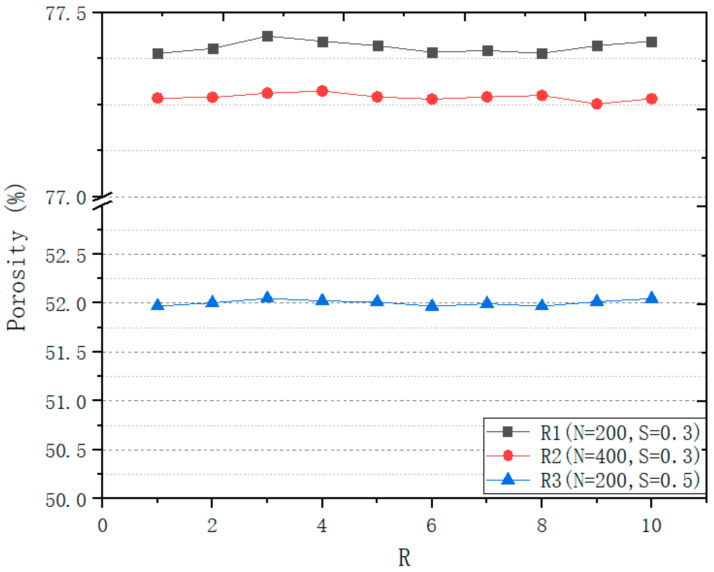
Relationship between *R* and porosity.

**Figure 15:**
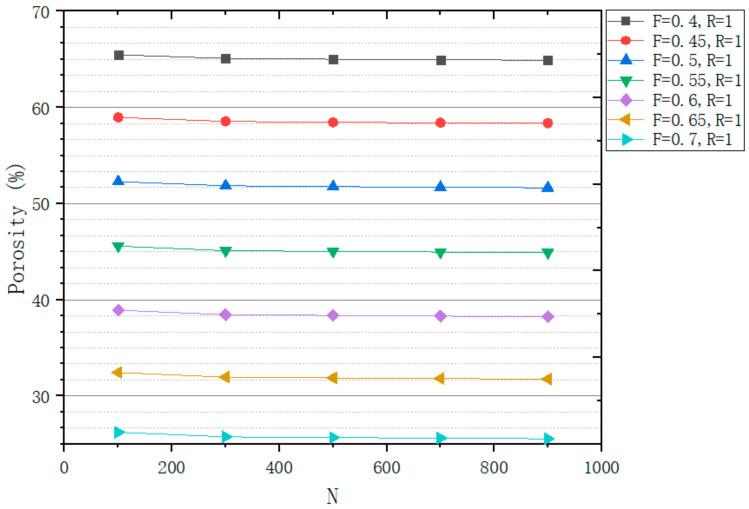
Relationship between *N* and porosity.

**Figure 16:**
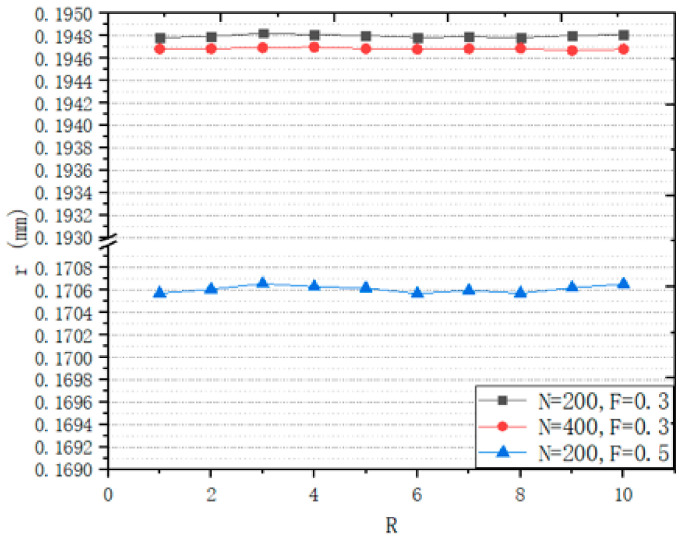
Relationship between *R* and r.

**Figure 17:**
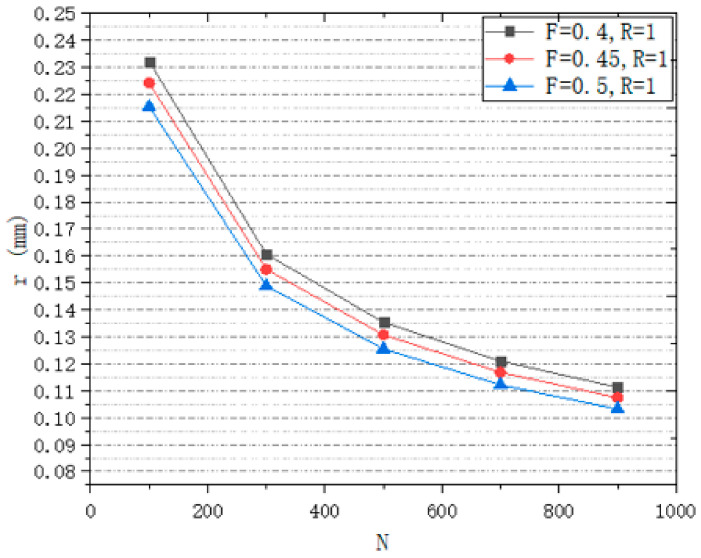
Relationship between *N* and r.

In order to accurately control the design of the structure, this work has studied the influence of design variables on the structure. This work designs a series of structures to study the influence of different variables on structural design. A design space of a box of 8 mm^3^ is generated to study the relationship between *R* and porosity (Figure 14), the relationship between *N* and porosity (Figure 15), the relationship between *R* and r (Figure 16), and the relationship between *N* and r (Figure 17).

As Figure 14 and Figure 15 shows, the effect of *N* and *R* on porosity is very small. However, the effect of *F* on porosity is decisive. So, the study of the effect of *F* on the porosity is the key to accurately control the porosity of the structure.

From Figure 16, it can be seen that with the change of *R*, the change of *R* is less than 0.2 μm. The effect of *R* on r can be ignored. We can see from Figure 17, with the increase in *N*, r decreases. *N* is estimated by Formula (5) and the relationship between *N* and r of actual structure is consistent with the Formula (5).

**Figure 18:**
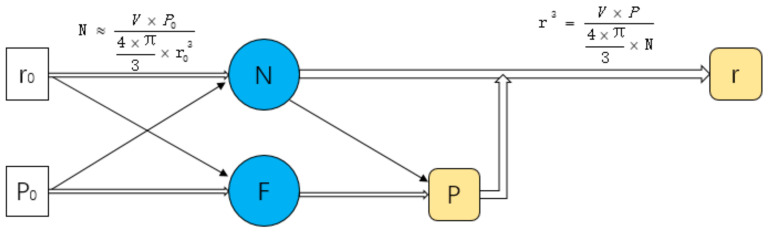
The relationship of the parameters.

However, *F* value has an effect on r. For a scaffold with an average pore radius of 200 μm, 20 μm is a big difference. In order to get the target structure accurately, the relationship between variables is studied. Suppose that the target porosity of a part of the scaffold is P_0_ and the target average pore radius is r_0_, and the actual porosity of the scaffold is P and the actual average pore radius is r. The relationship between them is shown in Figure 18.

**Figure 19:**
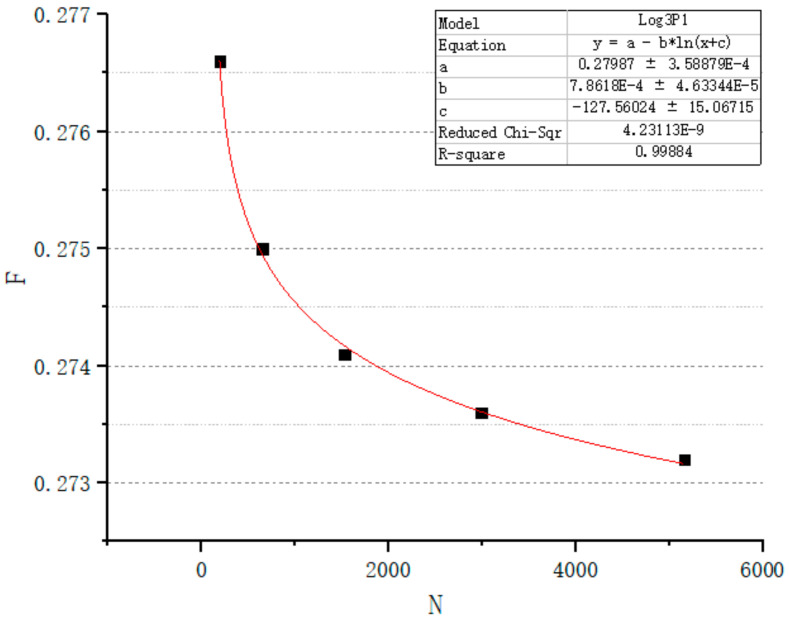
Relation between N and actual F.

**Figure 20:**
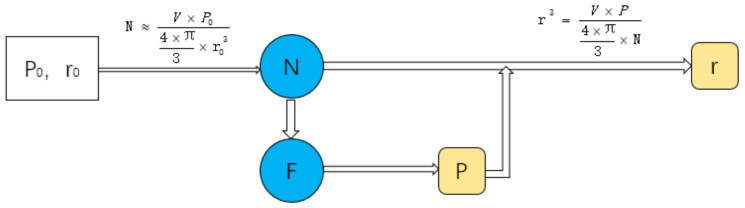
The relationship of the parameters after simplification.

In order to simplify the design process and simplify the relationship between design variables and design targets, the relationship between *F* and *N* is studied. Facing a certain target P and target r, we set several design spaces (Table 3). The target r is 200 μm. The target P is 80%. The number of target seeds is calculated. Adjust *F* to make the structure reach the target porosity. Draw a graph to show the relationship between *N* and the actual *F* and get the fitting curve (Figure 19). In this study, Log_3_P1 model was used as the equation of fitting curve, and the value of R-square was 0.999. Therefore, after determining the target porosity and target average pore radius, this formula can be used to predict the actual *F* value. So, the relationship between parameters can be simplified as seen in Figure 20.

The above design space is small. The real design space is much larger. According to our experience in the research process, the increase in design space will make the calculation time increase exponentially. Since the relationship between *F* and *N* has been obtained by fitting formula, the *F* of the target structure with larger design space can be predicted. Then we verified it in the design space of 1000 mm^3^.

The formula is used to predict the actual *F* of the structure with volume of 1000 mm^3^. The target porosity is 80% and the target pore radius is 0.2 mm. According to Formula (1), seed number *N* is calculated as 23874. The calculated *F* is 0.271949 according to the formula obtained in Figure 18. Then, the structure is designed with *F* as 0.271949. As a result, the actual porosity of the structure is 80.05%. The actual average pore radius is 0.20003954 mm. It is feasible to use this formula to predict F.

It can be seen from Figure 14 and Figure 16 that the *F* values corresponding to different targets P and r will be different. Though the Log3P1 fitting model is also applicable for different targets P and r, it should be noted that for different targets P and r, different specific formulas need to be obtained. Although it is also possible to manually try *F* to obtain the target P and r, the structure must be recreated for every *F* value. According to our research experience, when the design space is 216 mm^3^, the time required to generate the structure is only a few minutes. However, when the design space is 1000 mm^3^, the time will exceed 50 min. The complexity of the porous structure will consume a lot of computing time and computing resources, especially when the design space exceeds 1000 mm^3^. Therefore, we believe that it is very meaningful to obtain a specific formula through small design spaces, and then predict an accurate *F*.

Combining these control methods, the Voronoi porous structure scaffold that can match the natural bone pore structure and elastic modulus can be designed and generated. The biomimetic bone tissue engineering scaffold which can reduce stress shielding has been realized. Then porous structures can be manufactured with high precision through AM [18,19].

#### 3.1.2. Seeds Control Analysis

Seed distribution control can make the pore structure of the scaffold similar to the pore structure of natural bone. Based on the research in Section 2.1.1 and Section 3.1.1, different pore distribution can be realized by controlling the seed distribution.

**Figure 21:**
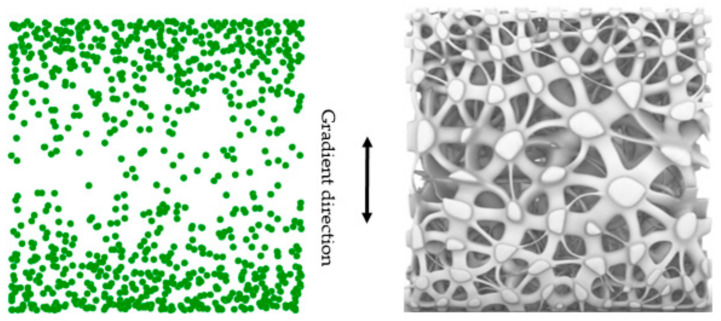
Structure with gradient seed and pore size in Z direction.

**Figure 22:**
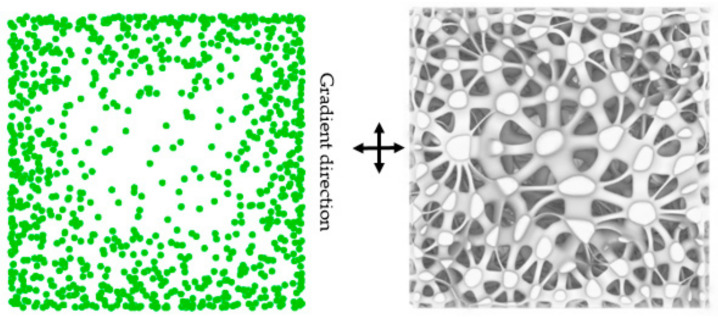
Structure with gradient seed and pore size imitating bone.

We make a simple gradient distribution control to make the number and size of pores gradient along the Z direction (Figure 21). The pore distribution of natural bone is generally sparse in the middle and dense near the edge. Then we imitate the pore structure of bone and control the pore distribution (Figure 22).

#### 3.1.3. Porosity Control Analysis

The porosity of the structure affects the apparent elastic modulus of the structure [18,19]. The target porosity can be obtained accurately and quickly through the fitting formula obtained in Section 3.1.1. By reasonably controlling the data structure in Grasshopper and adding the “loft” module, we can control the apparent elastic modulus of different parts of the structure according to the target requirements.

**Figure 23:**
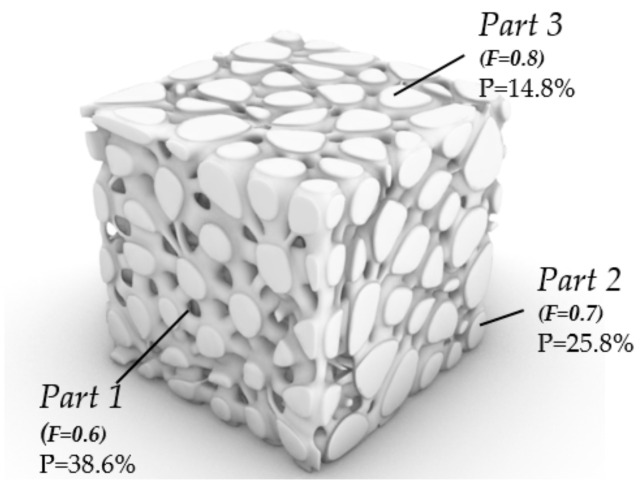
Structure with different porosity in different parts.

To verify the porosity control method studied in this paper, different surfaces with different porosity are designed (Figure 23).

**Figure 24:**
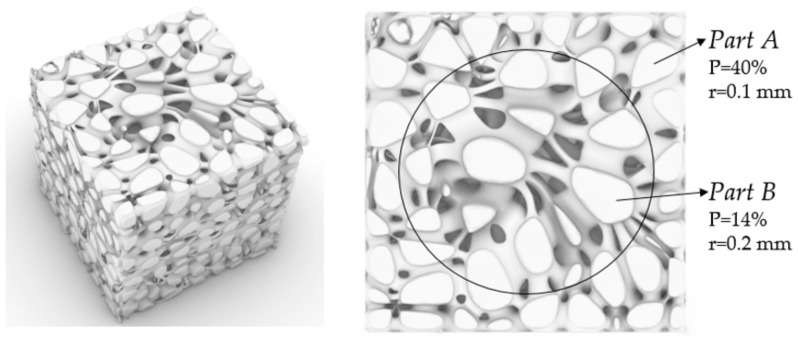
A gradient structure with gradient porosity and pores.

The goal of bionic design is to imitate natural bone with gradient pore and porosity. Therefore, we generated a simple structure with gradient pore and gradient porosity as verification (Figure 24). The volume of the box is 27 mm^3^. The design space of part A is 27 mm^3^ to 3π mm^3^. The design space of part B is 3π mm^3^.

### 3.2. Additive Manufacturing of Structure

**Figure 25:**
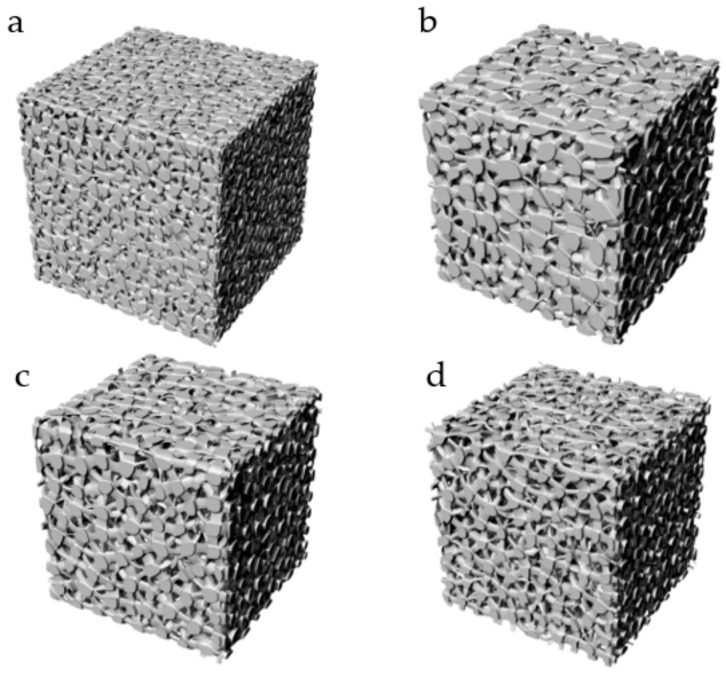
Structures with target porosity and target average pore radius. (**a**) Structure with P = 40%, r = 0.3 mm; (**b**) Structure with P = 40%, r = 0.5 mm; (**c**) Structure with P = 50%, r = 0.5 mm; (**d**) Structure with P = 60%, r = 0.5 mm.

**Figure 26:**
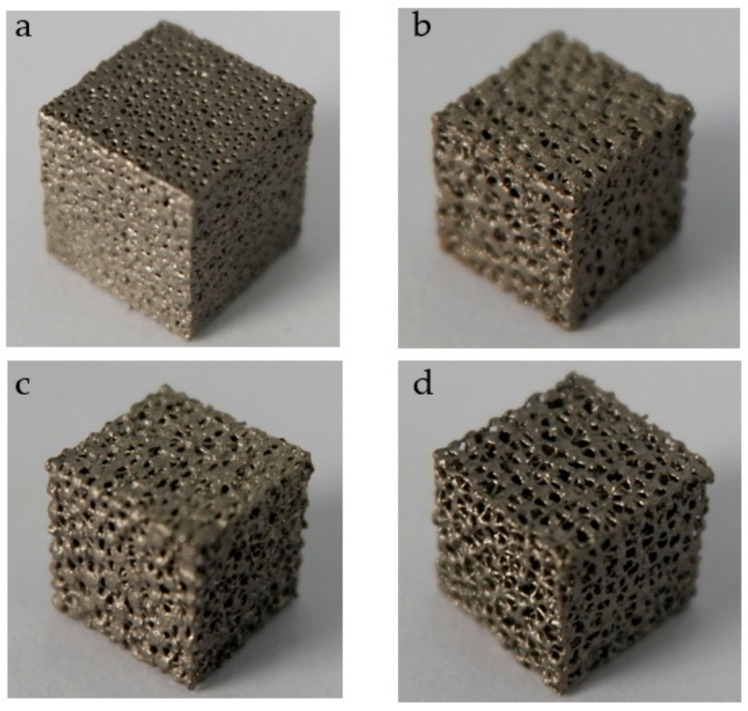
Ti6Al4V structures with target porosity and target average pore radius made by SLM. (**a**) Structure with P = 40%, r = 0.3 mm; (**b**) Structure with P = 40%, r = 0.5 mm; (**c**) Structure with P = 50%, r = 0.5 mm; (**d**) Structure with P = 60%, r = 0.5 mm.

According to the design method, we design a series of structures to verify the feasibility. Firstly, a group of structures with a certain target porosity and target average pore radius are designed (Table 4, Figure 25). In this work, in order to verify the manufacturability of the designed structure, several models are manufactured by means of the SLM (Selective laser melting) under equipment EOS M290 (Manufactured by Tolerance, Shanghai, China) (Figure 25, Figure 26). The manufacturing material is Ti6Al4V powder with a diameter of 30–45 μm.

**Figure 27:**
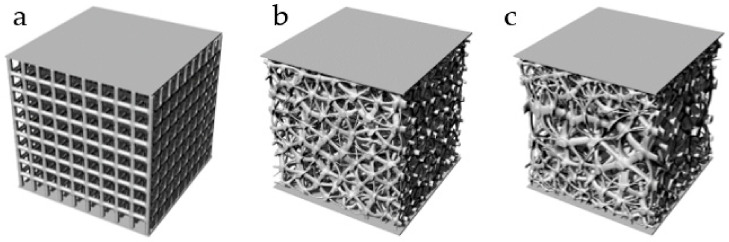
Designed models: (**a**) Regular porous structure; (**b**) Irregular porous structure; (**c**) Gradient irregular porous structure.

**Figure 28:**
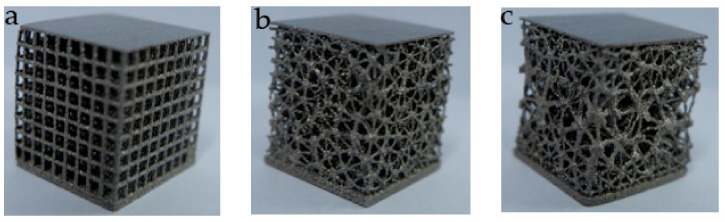
Ti6Al4V structures made by EDM. (**a**) Regular porous structure; (**b**) Irregular porous structure; (**c**) Gradient irregular porous structure.

Three kinds of structures with the same volume of 1784 mm^3^ and pore number of 729 are designed at the same time (Table 5, Figure 27). They were manufactured by means of the EBM (Electron Beam Melting) under printing equipment Arcam Q10 (Manufactured by Arcam, Sweden) (Figure 28). The manufacturing material is Ti6Al4V powder with a diameter of 45–60 μm. They are about to be models to verify stability performance in Section 3.3 of this paper.

It can be seen that the roughness of the structure made by SLM is better than that made by EBM. The good thing is both structures can be manufactured by SLM and EBM without internal support. Due to the lack of time and experimental equipment, what is the difference in strength and other mechanical properties between structures manufactured by SLM and EBM is not done. Manufacturing accuracy has not been evaluated. The good thing is that this work has verified that the structure obtained by this structural design method can be manufactured.

### 3.3. Stability Performance

#### 3.3.1. FEA of Stability Performance

The FEA was carried out in the finite element analysis software ABAQUS 2018 (Dassault Systemes SIMULIA, USA) according to the previously recommended method. We expected that after the force is applied in the vertical direction, the average value of displacements of structure 1 should be smaller than that of the other two structures, and the stability under force will be worse. However, it turns out that what we expected is not quite right.

**Figure 29:**
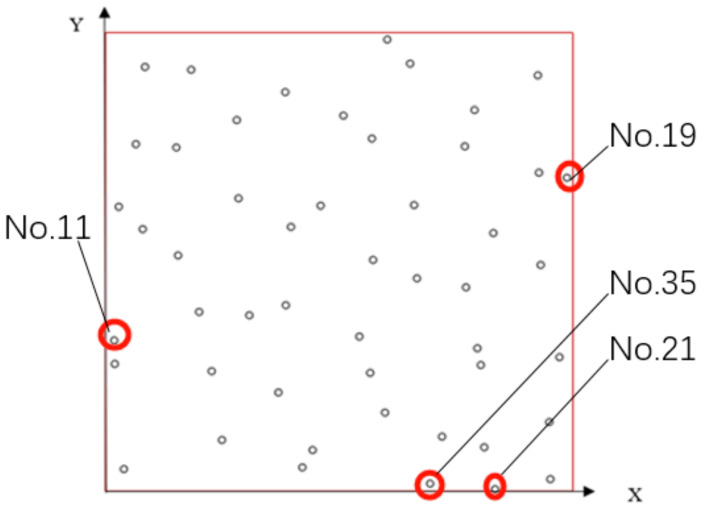
Points where the vertical displacements of the thin plate are larger.

Each FEA was performed once when force was applied to different points. Finally, 50 vertical displacements of 50 points of the three structures are obtained when 50 random points were subjected to a force of 50 N. We can see that at each different point, the displacement of each point is different. The displacement of the points near the corners is larger, such as point 11,19,21,35 (Figure 29). In our analysis, this is because there is not enough support at the corners, which makes the displacement of the thin plate larger.

**Figure 30:**
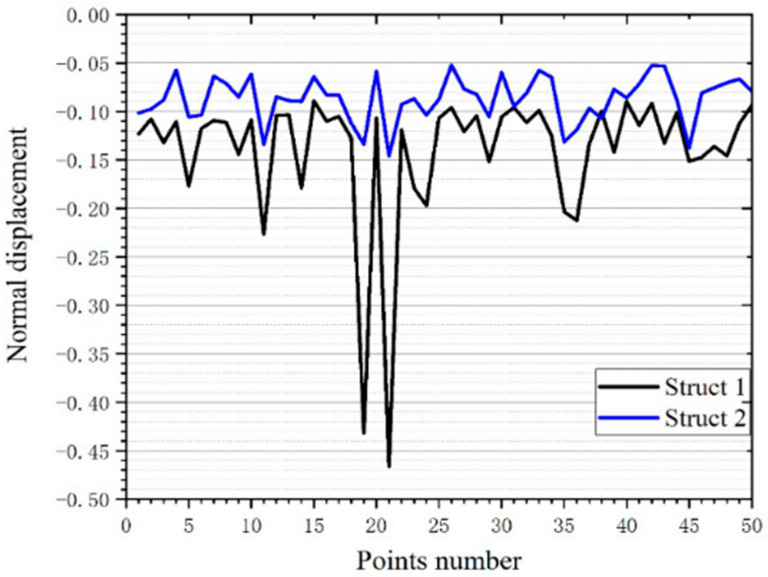
Comparison of the normal displacement of the force point of structure 1 and structure 2.

The FEA of the three structures can be divided into two control groups. The first group is the comparison of structure 1 and 2, namely the comparison of regular structure and irregular structure (Figure 30). Through statistical calculation, the displacement variance, and the displacement average value of the two sets of data are obtained. The difference in variance is considerable. The displacement variance of structure 1 is much larger than that of structure 2. From this we can easily judge that the stability of the irregular structure is obviously better when it receives the same magnitude of directional force.

The positions of the points in this work are randomly generated. It can be imagined that no matter where the point is, it will not cause excessive deformation due to the change of the action position when scaffold with this structure is under force. The whole scaffold surface has good anti deformation ability. The contact between human natural bone and scaffold is not completely fit. So, this FEA also simulates the actual stress situation of scaffold to a certain extent. Due to the different contact position with the scaffold, this stability makes the scaffold have no excessive displacement when contacting with natural bone. We think that this can reduce the wear of the bracket and improve its service life. This is undoubtedly beneficial.

In addition, we can see from the average displacement that the average displacement of structure 2 is smaller than that of structure 1, which is contrary to what we expected before. This can indicate that: when the scaffold is subjected to the force from bone, the looseness will be less, and the wear will be less. After the force is removed, the scaffold will fit better.

**Figure 31:**
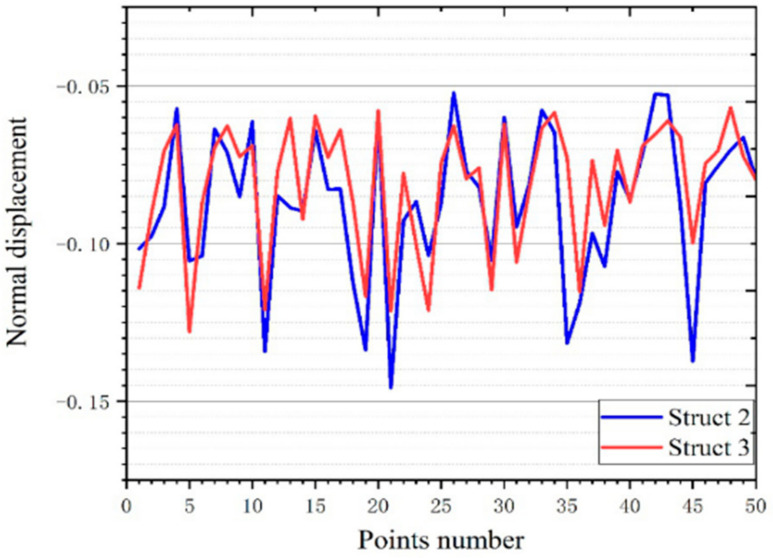
Comparison of the normal displacement of the force point of structure 2 and structure 3.

We speculate that this is an excellent performance brought about by more support rods closed to the thin plate. To verify this speculation, we did verification. The comparison of structure 2 and structure 3, namely the comparison of non-gradient structure and gradient structure (Figure 31). The result is as we speculated. It can be seen that the average displacement of structure 3 is smaller than structure 2 (Table 5). In addition, the displacement variance of structure 3 is smaller than that of structure 2. This is pleasing. This proves that more support near the thin plate can improve the stability of the scaffold.

#### 3.3.2. Experimental Verification of Stability Performance

**Figure 32:**
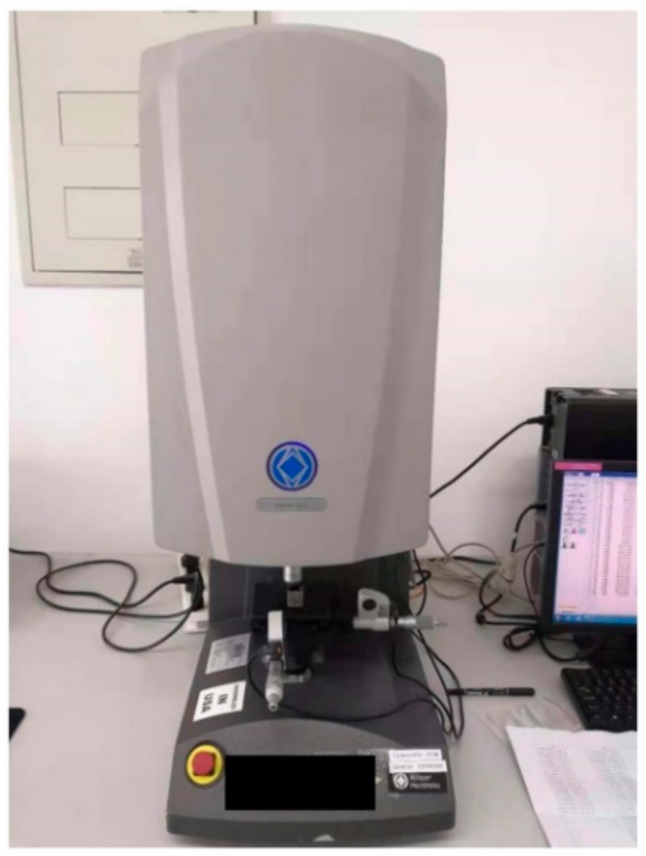
Vickers hardness testing system TUKON2500.

**Figure 33:**
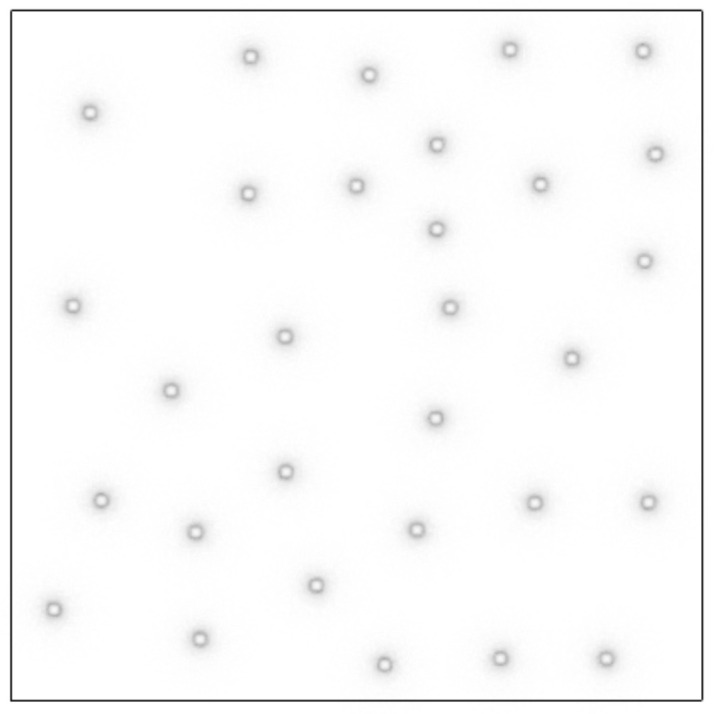
The position of 30 random points on the top plate of the structure.

**Figure 34:**
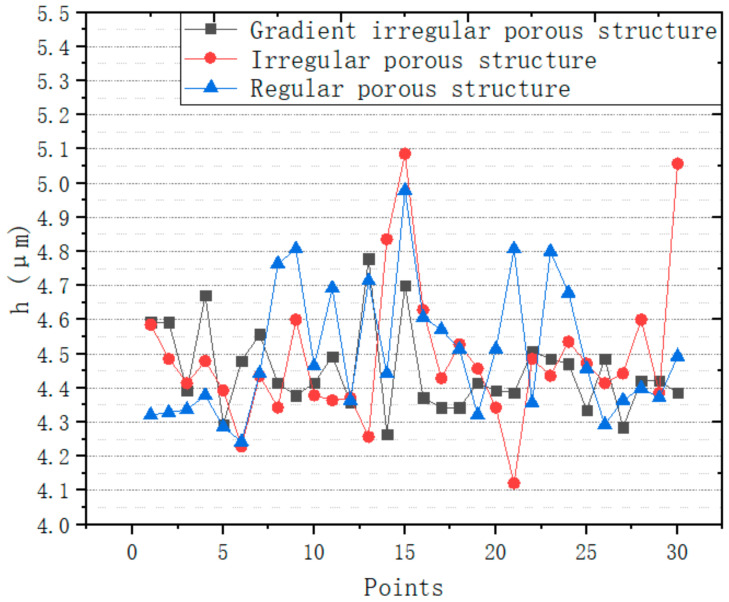
Indentation depth at 30 different points.

The experiment was carried out in the Vickers hardness testing system TUKON2500 (Manufactured by Wilson Hardness, USA) (Figure 32). The experiment meets the standard of ISO 6507-1:2005. The experimental model has been completed in Section 3.2 (Figure 28). The three models have the same design space of 800 mm^3^, the same mass of 10.5 g, the same pore number of 729. To verify the simulation results, 30 points are randomly generated on the top plate of the structure (Figure 33). The experimental load is 0.2 kg. After loading, the average diagonal length *d* of the indentation is counted. According to ISO 6507-1:2005, the indentation depth h is calculated by Formula (5). The experimental data were plotted, and the mean value and variance value were calculated (Figure 34, Table 6).

(6) h=d/7

The average value and variance value of indentation depth of the three structures have obvious changes. The average value and variance value of indentation depth of gradient irregular porous structure, irregular porous structure and regular porous structure decrease in turn. From the experimental results, it can be concluded that the stability of irregular porous structure is better than that of regular porous structure. The structure with gradient irregular porous structure has better stability performance. Therefore, the results of FEA are verified.

### 3.4. Impact Resistance Performance

#### 3.4.1. Deformation Displacement 

**Figure 35:**
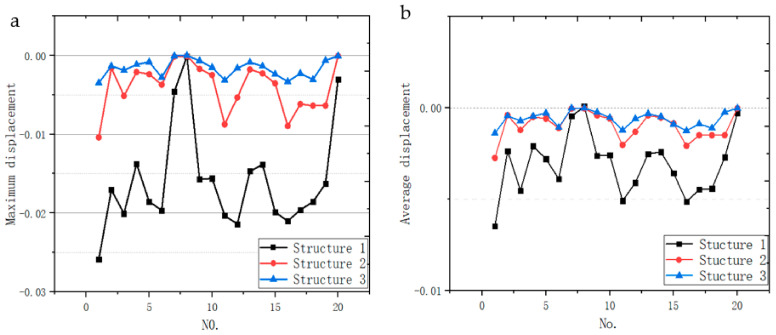
Comparison of displacements of bottom points of three structures under impact: (**a**) Maximum displacements; (**b**) Average displacements.

Through FEA, we collected the displacements of all points of the bottom of three kinds of plates after being impacted. In order to analyze the impact resistance of the structure more comprehensively, the maximum and average displacement of all points of the bottom are taken as the analysis objects. In total, 20 different points represent 20 different impact positions, so 20 sets of data are collected. The maximum and average displacements of 20 groups of points are obtained by calculation (Table 7). It can be clearly seen from Figure 35. The maximum displacement and average displacement of structure 1 are obviously larger than those of the other two irregular porous structures. From this point of view, it is proved that the impact resistance of regular structure is the worst. Then, two irregular porous structures were compared. The two displacement values of structure 3 are minimum. This shows that the impact resistance of the scaffold with gradient porous structure is better than that of the scaffold with non-gradient structure.

#### 3.4.2. Energy Absorption

**Figure 36:**
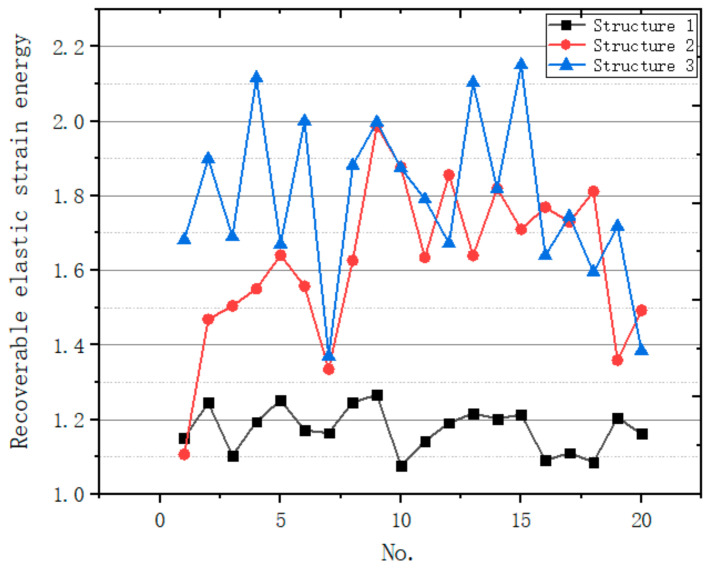
Comparison of recoverable elastic strain energy of three structures.

The external impact performed on three structures is the same, and the energy brought by the impact is converted into the internal energy, viscous dissipation energy, friction dissipation energy and kinetic energy of the structure. The internal energy is mainly converted into the recoverable elastic strain energy of the structure. Compared with internal energy, other forms of energy can be ignored. The recoverable elastic strain energy of three kinds of structures under different impact forces is calculated (Figure 36).

The recoverable elastic strain energy of structure 3 is the largest, that of structure 2 is the second, and that of structure 1 is the smallest. So, compared with regular porous structure, Voronoi porous structure can absorb more energy, then turn it into recoverable elastic strain energy. Gradient Voronoi porous structure performs better. The recoverable elastic strain energy inside the scaffold is large, so most of the energy is absorbed by the structure. This can also explain why the displacement of the bottom surface of the gradient porous structure is the smallest after the impact.

For bone scaffolds, good impact absorption performance can make the scaffolds absorb the energy from healthy bones and transform it into recoverable elastic strain in the structure. The growth of new bone tissue in bone tissue engineering scaffolds will be accelerated under certain mechanical stimulation [27,28]. The volume and mass of the three structures are the same in our work. With the same volume and mass of the structure, more recoverable elastic strain represents the mechanical stimulation generated inside the structure, which is believed to be beneficial to the growth of new bone tissue [27,28,29]. Moreover, the impact on the support is absorbed by the gradient Voronoi porous structure, which makes the deformation displacement of the bottom surface of the structure smaller. When the gradient porous structure is applied to scaffolds and bone graft materials, it will certainly bring less loosening with natural bone, which is also a beneficial performance.

Many bones (such as the tibia) bear forces and transmit forces with a certain impact in human activities. Then, when the scaffold with irregular porous structure bears the impact from natural bone, it can have a better impact resistance effect. The anti-impact scaffold can be equivalent to a buffer module, which can absorb the energy from the impact. This part of energy is dispersed in the supporting beams, which is beneficial to stimulate the growth of bone in the scaffold.

## 4. Conclusions

In order to reduce the stress shielding between the scaffold and bone and control the apparent elastic modulus of the scaffold, a structural design method based on Voronoi-tessellation method was proposed. This method can flexibly and accurately adjust the distribution of the pores and porosity of the scaffold. This design method further promotes the design of bone scaffolds that mimic natural bones. Apparent elastic modulus is related to porosity. Gradient porosity scaffolds are worth looking at to reduce stress shielding. Based on this structure design method, the scaffolds with irregular porous structure and gradient irregular porous structure were obtained. Several structure models are made by SLM and EBM. It is verified that the structure obtained by this design method can be manufactured.

In order to study the mechanical properties of irregular porous structure, FEA analysis was carried out. The results show that the irregular porous structure has better performance in stability and impact resistance than the regular structure. It is verified that the scaffold with gradient structure has better stability and impact resistance than the non-gradient structure. Then an experiment is carried out to prove the stability performance. A better performance in stability and impact resistance is beneficial to improve the mechanical properties of the scaffold, increase the service life of the scaffold and promote the growth and healing of the bone. We believe this study also provides a theoretical basis for the design of porous scaffold.

## Figures and Tables

**Table 1:** The coordinates of 50 random points on the thin plate.

No.	Coordinate	No.	Coordinate	No.	Coordinate
1	{4.168322, 0.018732}	21	{4.168322, 0.018732}	41	{2.847754, 3.846355}
2	{1.916102, 4.356278}	22	{1.916102, 4.356278}	42	{1.924213, 2.023893}
3	{4.761867, 0.12438}	23	{4.761867, 0.12438}	43	{2.860703, 2.517847}
4	{0.1905, 0.236104}	24	{0.1905, 0.236104}	44	{4.053635, 0.474514}
5	{4.624188, 4.534302}	25	{4.624188, 4.534302}	45	{0.137717, 3.099845}
6	{2.710723, 1.68053}	26	{2.710723, 1.68053}	46	{4.858988, 1.457464}
7	{4.655686, 2.465611}	27	{4.655686, 2.465611}	47	{2.986517, 0.852671}
8	{0.770162, 2.565942}	28	{0.770162, 2.565942}	48	{3.85815, 2.217037}
9	{2.10183, 0.251383}	29	{2.10183, 0.251383}	49	{0.999095, 1.950896}
10	{3.299956, 3.113036}	30	{3.299956, 3.113036}	50	{1.845819, 1.070618}
11	{0.094996, 1.382577}	31	{3.256359, 4.662494}		
12	{3.948792, 4.156852}	32	{1.130099, 1.30636}		
13	{3.597641, 0.589451}	33	{1.977037, 2.874706}		
14	{2.211046, 0.441559}	34	{4.014768, 1.36867}		
15	{3.329278, 2.313848}	35	{3.4652, 0.079034}		
16	{2.538373, 4.098269}	36	{0.085785, 1.639095}		
17	{1.416367, 3.189073}	37	{0.755195, 3.747541}		
18	{0.322663, 3.78601}	38	{4.63717, 3.476797}		
19	{4.939515, 3.423519}	39	{3.847277, 3.762397}		
20	{3.980097, 1.554705}	40	{0.909925, 4.59761}		

**Table 2:** Coordinates of 20 random points for impact resistance.

No.	Coordinates	No.	Coordinates
1	{2.496821,2.501216,0}	11	{1.840198, 2.191361, 0}
2	{1.012634,1.123176,0}	12	{3.518079, 3.569665, 0}
3	{2.100246,3.506155,0}	13	{0.706907, 3.262782, 0}
4	{4.402913,1.523841,0}	14	{0.647487, 1.881901, 0}
5	{3.799921,4.100211,0}	15	{4.083439, 2.707272, 0}
6	{3.855469, 2.020813, 0}	16	{2.280039, 1.804911, 0}
7	{0.366727, 4.477109, 0}	17	{3.493572, 3.065361, 0}
8	{4.877725, 4.826198, 0}	18	{3.086525, 1.606976, 0}
9	{2.460168, 4.462378, 0}	19	{1.582868, 4.209732, 0}
10	{2.547036, 0.568136, 0}	20	{4.69689, 0.445073, 0}

**Table 3:** To achieve target P_0_ and target r_0_, adjust actual *F* to design structures with actual P and r.

V (mm^3^)	8	27	64	125	216
*N*	191	645	1528	2985	5157
*F*	0.2766	0.275	0.274	0.2736	0.2732
P	80.003%	80.005%	79.998%	80.004%	79.998%
r	0.199998	0.19996	0.199993	0.199984	0.199993

**Table 4:** Structures with target porosity and target average pore radius.

Structure	a	b	c	d
P	40%	40%	50%	60%
r (mm)	0.3	0.5	0.5	0.5

**Table 5:** Force analysis results.

	Volume (mm^3)^	Quality (10^–5^ kg)	Average Displacement (mm)	Variance
Structure 1	13.30	5.89	−0.14016	0.005063
Structure 2	13.30	5.89	−0.08699	0.000541
Structure 3	13.30	5.89	−0.08127	0.000403

**Table 6:** The mean value and variance value of h.

Structures	Mean Value (μm)	Variance Value
Irregular porous structure	4.526667	0.178685
Regular porous structure	4.505	0.203792
Gradient irregular porous structure	4.406238	0.111739

**Table 7:** Displacements of all points collected from the bottom of structures.

No.	Max Displacements			Ave Displacements		
	Structure 1	Structure 2	Structure 3	Structure 1	Structure 2	Structure 3
1	−0.025865313	−0.010407655	−0.00349243	−0.006474	−0.002738	−0.001383
2	−0.017054364	−0.001593933	−0.00134111	−0.002355	−0.000394	−0.000438
3	−0.02006232	−0.005133115	−0.00187291	−0.004522	−0.001203	−0.000708
4	−0.013765119	−0.002104803	−0.00115216	−0.002092	−0.000498	−0.000448
5	−0.018555669	−0.002373617	−0.00081490	−0.002789	−0.000586	−0.000278
6	−0.019666893	−0.003702455	−0.00278609	−0.003882	−0.001011	−0.001074
7	−0.004564771	−0.000147287	−4.5075 × 10^−8^	−0.000454	−3.2 × 10^−5^	−1.4 × 10^−5^
8	−0.000111257	−9.33554 × 10^−5^	−1.6733 × 10^−8^	−7.2 × 10^−5^	−2.2 × 10^−5^	−8.00 × 10^−5^
9	−0.015718957	−0.001704743	−0.00067792	−0.002621	−0.000411	−0.000238
10	−0.01560978	−0.002497677	−0.00150531	−0.002576	−0.000581	−0.000531
11	−0.020331565	−0.008744049	−0.00314237	−0.005078	−0.002024	−0.001216
12	−0.021405157	−0.005330172	−0.00161022	−0.004096	−0.001302	−0.000592
13	−0.014698667	−0.001771639	−0.00086958	−0.002514	−0.000412	−0.000316
14	−0.013848106	−0.002285997	−0.00134816	−0.002408	−0.000528	−0.000476
15	−0.019885683	−0.003542745	−0.00236954	−0.003574	−0.000854	−0.000891
16	−0.021027157	−0.008946069	−0.00331503	−0.005126	−0.002074	−0.001246
17	−0.019596884	−0.006173599	−0.00227502	−0.004471	−0.001495	−0.000874
18	−0.018581722	−0.006368216	−0.00305709	−0.004416	−0.006368	−0.001102
19	−0.016262516	−0.006368216	−0.00063789	−0.002713	−0.003461	−0.000228
20	−0.003053466	−5.70507 × 10^−5^	−4.6086 × 10^−5^	−0.000266	−1.3578 × 10^−5^	−1.434 × 10^−5^

## Data Availability

Data is contained within the article.
